# Large language models and artificial intelligence for generating, simplifying, and enhancing outpatient clinic letters: A systematic review

**DOI:** 10.1371/journal.pdig.0001745

**Published:** 2026-09-22

**Authors:** Rocco Sheldon, Morya Wadodkar, Andrew Gan, Rachael Yip, Yimeng Zhang, Jyoti Baharani

**Affiliations:** 1 College of Medicine and Health, University of Birmingham, Birmingham, United Kingdom; 2 Bristol Medical School, University of Bristol, Bristol, United Kingdom; 3 Department of Renal Medicine, University Hospitals Birmingham NHS Foundation Trust, Birmingham, United Kingdom; The University of Texas at Austin, UNITED STATES OF AMERICA

## Abstract

Outpatient clinic letters are a cornerstone of clinical communication but frequently exceed recommended reading levels, limiting patient comprehension and engagement. Large Language Models (LLMs) and Artificial Intelligence (AI) have been proposed as scalable tools for generating and simplifying these documents, but the evidence regarding their clinical accuracy, safety, and effectiveness remains fragmented. We conducted a systematic review of studies evaluating AI or LLMs for generating, simplifying, or enhancing outpatient clinic letters. Five databases (PubMed, EMBASE, Web of Science, CENTRAL, and CINAHL) were searched from inception to 1 November 2025. Two independent reviewers screened studies, extracted data, assessed quality using the Mixed Methods Appraisal Tool (MMAT), and certainty using the GRADE appraisal tool. Seven studies were included, comprising two studies using real-world clinic data and five using synthetic or hypothetical scenarios. Findings regarding readability were mixed; while some AI models improved readability scores compared to human-authored letters, most AI-generated content still failed to meet the recommended US Grade 6 reading level. The two studies that measured patient-reported outcomes reported high satisfaction and comprehension with AI-simplified letters, but these studies relied on subjective measures of understanding or lacked a human-authored control group. Clinical accuracy varied substantially, with information fidelity ranging from 10% to 100%. Risks of ‘hallucinations’, inappropriate medical advice, and tactless patient descriptions were documented. One study reported a ten-fold reduction in drafting time compared to human dictation. Overall certainty of evidence was rated very low across all primary outcomes due to methodological limitations, small sample sizes, and reliance on hypothetical scenarios. The current evidence supporting AI and LLMs use for outpatient clinic letters is preliminary and limited, providing insufficient evidence to support routine clinical implementation beyond supervised experimental and quality-improvement settings. Concerns regarding accuracy, safety, and generalisability remain. Further real-world patient-centred research is required before widespread adoption. PROSPERO registration: CRD420251181303.

## Author summary

Outpatient clinic letters are a central part of how doctors communicate with patients, yet they are routinely written at a reading level far above what most people can understand. Despite longstanding guidance in the United Kingdom recommending that letters be written directly to patients, this remains inconsistently practised: partly due to time pressures and concerns about losing clinical detail. Artificial intelligence (AI) tools, particularly large language models such as ChatGPT, have been proposed as a scalable solution to this problem; however, the evidence underpinning their use has not previously been synthesised. We conducted a systematic review of seven studies evaluating AI for writing, simplifying, or enhancing clinic letters. We found that preservation of clinical information was highly variable (10%-100%), and that while two studies reported positive patient satisfaction and understanding, these studies had significant limitations in their design. No AI tool consistently achieved recommended reading levels using formal readability measures. Risks of inaccurate or inappropriate hallucinations were documented across studies. Overall, the quality of the evidence was very low. We conclude that whilst promising, the current evidence does not support the routine use of AI for clinic letters beyond research settings, and that larger, real-world, patient-centred studies are urgently needed.

## Introduction

Outpatient clinic letters are a cornerstone of clinical communication, serving as a formal record of patient encounters and a key mechanism for information exchange between patients and their primary and secondary care physicians. These letters typically summarise diagnoses, investigations, management and follow-up plans, and play a critical role in continuity and safety of care [1]. These differ from clinic notes, which are generally shorthand internal records of an encounter.

Historically, outpatient letters have been written primarily for General Practitioners (GPs), with patients not routinely included in the communication loop. Over the past two decades, however, healthcare policy has increasingly emphasised transparency, shared decision-making, and patient autonomy. In the UK, the *NHS Plan* (2000) was an early catalyst for this change, recommending that all patients routinely receive copies of letters sent to their GPs [2]. This direction was reinforced by guidance from the Academy of Medical Royal Colleges (AoMRC) in 2018, which advocated that outpatient letters should be addressed directly to patients, rather than copied to them [3]. This guidance was formally endorsed by NHS England in 2023 [4]. Together, these changes reflect a broader shift towards patient-centred care and recognition of written communication as a tool for patient engagement, rather than solely professional correspondence.

Despite these policy changes, substantial evidence indicates that outpatient clinic letters remain poorly aligned with patients’ health literacy levels. Numerous studies across a range of specialties have consistently shown that outpatient letters are written at a readability level exceeding that recommended by the U.S. Department of Health and Human Services, namely, U.S. Grade 6 (equivalent to a reading age of 11–12 years) [5,6]. Furthermore, this U.S. recommended level exceeds the average reading age of the U.K (9-years-old), where no guidance regarding clinic letter readability levels exists [7]. Readability itself is defined as “the determination by systematic formulae of the reading comprehension level that a person must have to understand written materials” [8], and measured using formal readability tools (e.g., Flesch Reading Ease and Flesch-Kincaid Grade Level) [9].

The benefits of simplified health communication extend beyond comprehension alone, with benefits to health outcomes well documented [10,11]. Despite this, a systematic review of 29,424 materials across 438 studies from 1990 to 2022 revealed that complexity of information in all clinical areas exceeded recommended levels for patients [9]. Although there is limited evidence on patient opinions regarding the complexity of clinic letters, dense, clinician-oriented language may hinder patients’ understanding, thereby limiting the effectiveness of shared decision-making and reducing the overall impact of the communication [12,13].

Several interventions have attempted to address the complexity of outpatient letters, including seminar-based teaching sessions and paper-based prompts. [14,15].

However, the results have been inconsistent, and a frequently cited challenge is the difficulty of simplifying language without losing clinical detail required by GPs [14]. One study which demonstrated the greatest improvement in readability was writing letters directly to patients [16]. In this study, clinicians dictated two separate letters for 84 patients: one to the patient’s GP and another written directly to the patient. Compared to GP-directed letters, patient-directed letters showed a significant improvement in the Flesch Reading ease score (P < 0.001), and a significant reduction in the usage of terms that patients didn’t understand (P < 0.001) [16].

While these findings are promising, concerns persist regarding time constraints and potentially insufficient clinical details in patient-directed letters [16,17]. Furthermore, a study of 107 community mental health clinic letters (including 49 patient-directed letters) found that all letters exceeded the recommended readability level of U.S. Grade 6 (reading age 11–12 years), and 63% exceeded the level expected of a 15-year-old [17]. These findings suggest that simply instructing clinicians to write directly to patients may be both impractical and insufficient to achieve meaningful improvements in health literacy.

The emergence of Artificial Intelligence (AI), particularly Large Language Models (LLMs) like ChatGPT, offers a potential novel, scalable technological solution to these long-standing challenges. Early studies suggest that AI tools may be able to rapidly regenerate existing clinic letters at similar or reduced complexity compared to clinician-authored letters, while preserving clinical accuracy [18,19]. AI-assisted approaches offer several potential advantages: 1) automation may reduce clinician workload and time spent on documentation; 2) AI tools could generate dual letters: one meeting recommended literacy levels for patients, and another preserving clinical detail for GPs; 3) AI may support training of resident doctors by modelling simplified communication styles; and 4) AI implementation is scalable across healthcare systems.

However, the current evidence base for AI-assisted clinic letters is limited and fragmented. Published studies are often small, confined to single specialties, and use heterogenous outcome measures [18]. Significant concerns remain regarding clinical accuracy, the risk of AI “hallucination” (generation of plausible but false information), ethical governance, data security, and patient safety. Without systematic synthesis, it remains unclear whether AI-generated clinical letters are reliable, effective, and appropriate for widespread clinical adoption.

Therefore, this systematic review aims to synthesise all available evidence on the use of AI and LLMs in the generation, simplification, and enhancement of outpatient clinic letters. By evaluating clinical accuracy, readability, patient comprehension, and safety outcomes, this review seeks to inform clinical practice and guide the design of future research in this rapidly evolving field.

## Methods

### Protocol registration

This systematic review was prospectively registered in the International Prospective Register of Systematic Reviews (PROSPERO) database as CRD420251181303 (available from https://www.crd.york.ac.uk/PROSPERO/view/CRD420251181303). The protocol was conducted in accordance with the Preferred Reporting Items for Systematic Reviews and Meta-Analyses (PRISMA) guidelines (S1 Table) [20].

### Search strategy

A systematic literature search was conducted across five databases: Embase (through Ovid), MEDLINE (through PubMed), Cochrane Central Register of Controlled Trials (CENTRAL), Cumulative Index to Nursing and Allied Health Literature (CINAHL), and Web of Science. A comprehensive search query was developed to identify all relevant publications investigating the use of AI or LLMs to generate, simplify, or enhance outpatient clinic letters, from database inception to 1 November 2025. Examples of search terms include “clinic letter”, “outpatient correspondence”, “patient communication”, “artificial intelligence”, “large language model”, “ChatGPT”, “Claude”, “Copilot”, “Grok”, “simplify”, “readability”, “accuracy”, “enhancement”, “Flesch-Kincaid”, “Flesch Reading Ease”. The full search queries for each database are presented in S2 Table. Both forward (looking through all the articles that cite papers included in the review) and backward (reference list checking) citation searches were performed to identify any additional studies.

### Study selection

Studies were screened against the following inclusion criteria defined using the PICOS framework. Population (P) included studies of outpatient letters in any medical or surgical specialty. Intervention (I) included the use of AI or LLMs to generate, modify, or simplify these letters. A Control (C) group was not required. Outcomes (O) must include at least one of clinical accuracy, readability metrics, patient comprehension, time efficiency, patient satisfaction, or compliance with clinical guidelines. All Study designs (S) were included. Exclusion criteria included referral letters, radiological reports, electronic health record summaries, discharge summaries, and any form of clinical communication not intended for patient consumption. Where only conference abstracts were available, corresponding authors were contacted to request additional information when contact details could be identified. No corresponding authors responded to our requests; therefore, these abstracts were excluded due to insufficient information. Preprints were eligible for inclusion, but no relevant preprint articles were identified after screening.

Title and abstract screening, followed by full text review, was conducted independently by two reviewers (RS and MW) using Covidence, with conflicts resolved by a third reviewer (AG).

### Data extraction

Data extraction and quality assessment were conducted by two independent reviewers (MW and RY), with conflicts resolved by a third reviewer (RS). The standardised data extraction spreadsheet covered the following domains: first author’s name, year of publication, study design, country, setting, specialty, sample size, AI/LLM tool, prompting strategy, target reading level, control group type (if applicable), source of prompts (e.g., hypothetical scenarios, anonymised real letters), and outcome measures.

Primary outcomes included clinical accuracy (assessed by error rates, omission of critical information, or clinician-determined accuracy scores), readability (measured using validated tools such as Flesch Reading Ease (FRE), Flesch-Kincaid Grade Level (FKGL), Gunning Fog index, or Simple Measure of Gobbledygook (SMOG) index), and patient comprehension (assessed through subjective Likert scores or formal assessment tools). Due to the heterogeneity in reporting of accuracy and safety outcomes, these have been mapped to three domains to assist interpretation: information fidelity (preservation/omissions of information), hallucinations (insertion of factually incorrect information), and qualitative clinical risk (including inappropriate assurance and tactless advice). However, heterogeneity remains within these three groups and analysis relies on the reporting of the primary studies. Secondary outcomes included time efficiency, patient satisfaction, healthcare professional satisfaction, and any noted barriers to implementation (S3 Table).

### Quality assessment

Given the anticipated heterogeneity in study designs, the relevant Mixed Methods Appraisal Tool (MMAT) was selected to assess the quality of included studies [21]. The tool assesses methodological quality within five categories: qualitative research, randomised controlled trials, non-randomised studies, quantitative descriptive studies, and mixed-methods studies.

### Data analysis

Given the limited number of included studies and heterogeneity in study designs, interventions, and outcome measures, quantitative meta-analysis was not feasible. Consequently, findings are tabulated and presented as a structured narrative synthesis, summarising and comparing results across AI/LLM model and outcomes reported. The GRADE criteria were used to assess certainty for each primary outcome [22].

## Results

**Fig 1:**
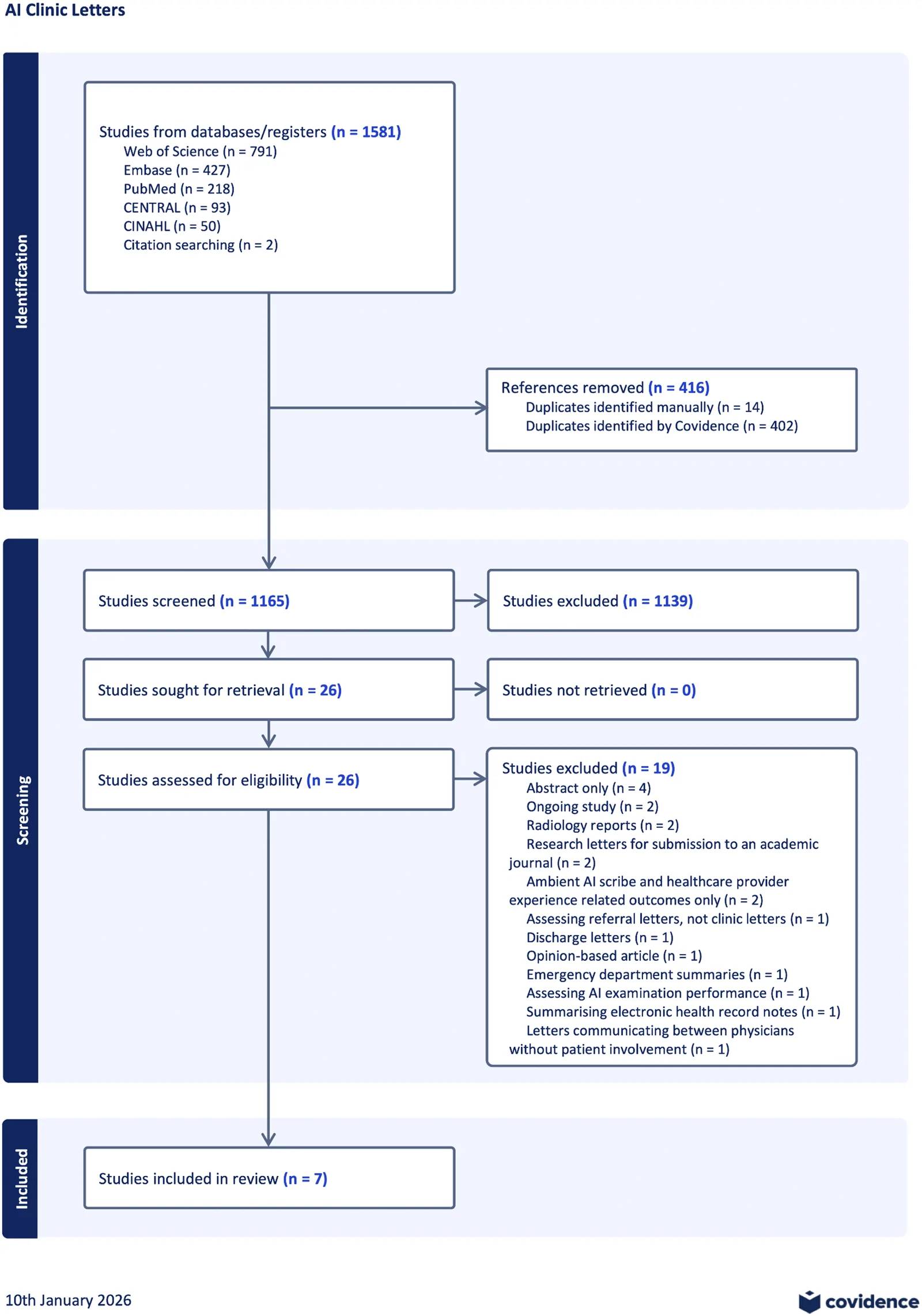
PRISMA flow diagram. CENTRAL, Cochrane Central Register of Controlled Trials; CINAHL, Cumulative Index to Nursing and Allied Health Literature.

The literature search across five databases and citation searching produced 1581 results. After removal of 416 duplicates, 1165 titles and abstracts were screened. Of these, 1139 were excluded, leaving 26 studies for full text screening. S4 Table lists all excluded studies with reasons. 19 studies were excluded for reasons outlined in the PRISMA flow diagram (Fig 1). This left seven eligible studies for data extraction. Study characteristics and results are summarised in Table 1.

**Table 1:** Detailed study characteristics and outcomes.

Study ID, Country	Sample & Setting	AI Intervention & Prompt Strategy	Control/ Comparator	Readability Outcomes	Patient- or Clinician-Centred Outcomes	Clinical Accuracy & Safety
Ali et al. (2023) UK	**n = 38** hypothetical scenarios **Setting:** Dermatology (Skin Cancer: 7 BCC, 11 SCC, 20 MM)	**Model:** ChatGPT-3.5 **Prompt:** Few-shot (increasing complexity) **Task:** Generate formal clinic letters based on clinical scenarios	**Comparator:** None **Design:** Descriptive simulation study	**Results:** FKGL Grade 9 (Reading age 14–15 years) **Target:** Grade 6 (Not met)	**Not assessed**	**Information fidelity:** 70% median (Range: 10–90%) **Hallucinations:** Risk of hallucinations noted in some outputs, but poorly described **Qualitative clinical risk:** 70% median humanness (range 50–90%) (clinician-reported 10-point Likert scale)
Shahnam et al. (2025) Australia	**n = 25** synthetic cases **Setting:** Medical Oncology (6 types: Lung, H&N, Colorectal, GU, Breast, Gynae) **Note:** Derived from 6 oncologists; 4 training cases	**Model:** ChatGPT-4o **Prompt:** Iterative (4 training cases used for refinement) **Task:** Generate first-visit consultation letters from synthetic notes	**Comparator:** None **Design:** Descriptive simulation study	**Results:** FRE 73.3 (range 63.1-83.4, Grade 7); SMOG 8.1 (range 7.1-9.1, Grade 8) **Finding:** AI text was significantly simpler than clinician control	**Patient-reported outcomes:** PEMAT-P (Understandability) >80% for AI-generated letters 72% of consumers willing to receive AI-generated letters based on examples, but no clinician-author comparison was provided	**Information fidelity:** Patient-directed letters: 80%; Clinician-directed letters 92–98%; Occasional omissions; iterative prompt refinement helped **Hallucinations:** Not reported **Qualitative clinical risk:** Some patients reported AI-generated letters to be robotic and less empathetic
Cork & Hopcroft (2025) UK	**n = 23** real consultant letters **Setting:** Multi-specialty (8 departments: ENT 6, Paediatrics 6, Gastroenterology 4, Neurology 2, Psychiatry 2, Renal 1, Gynaecology 1, Respiratory 1)	**Model:** ChatGPT-4 **Prompt:** Zero-shot (structured) **Task:** Simplify existing letters for average UK reading ability	**Comparator:** Original clinician-authored letters **Design:** Paired before-after study	**Results:** Gunning Fog: 11.35 (AI) vs 12.44 (Control; p = 0.02); Reading age: 16 years (AI) vs 17 years (Control; p = 0.15) Non-significant changes in FKGL, SMOG, Coleman-Liau, and automated readability scores	**Patient-reported outcomes:** Understanding of Diagnosis (4.41 vs 2.61); Understanding of Management (4.30 vs 2.78) Significant improvement for AI vs Control (p < 0.0001 for all measures) **Satisfaction:** 4.46 ± 0.20 (AI) vs 3.17 ± 0.62 (Control); p < 0.0001 **Preference:** 100% of patient representatives preferred the AI-simplified version	**Information fidelity:** 100%; all key clinical details retained **Hallucinations:** Zero hallucinations reported **Qualitative clinical risks:** Patients significantly preferred AI-generate letter language; no safety concerns identified by reviewing clinicians
Caterson et al. (2024) UK	**n = 15** simulated scenarios **Setting:** Orthopaedic Surgery (4 elective, 11 fracture cases)	**Models:** ChatGPT-4 and ChatGPT-3 **Prompt:** Zero-shot (single prompt) **Task:** Generate clinic letters from clinic note format	**Comparator:** AI vs AI comparison; No human control **Design:** Comparative simulation study	**Results:** ChatGPT -4: FRE 58.2 ± 4.00; FKGL 8.77 ± 0.918; Reading age 14–15 years ChatGPT-3: FRE 59.3 ± 6.98; FKGL 8.47 ± 0.982; Reading age 14–15 years **Target:** Grade 6 (Not met by either model)	**Not assessed**	**Clinician-reported accuracy:** Letter: ChatGPT-4 (87%) vs ChatGPT-3 (73%); p = 0.024 Management plan: ChatGPT-4 (79%) vs ChatGPT-3 (68%); p < 0.001 **Hallucinations:** Both models added unprompted and sometimes inaccurate details (e.g., specific cast removal dates) **Qualitative clinical risk:** Tactless descriptions of patients’ body weight
Stoneham et al. (2024) UK	**n = 70** real NHS hand clinic letters **Setting:** Hand Surgery (2 hospital sites) **Note:** 4-week period; new patients only. Clinicians: 27 Consultants, 24 Fellows, 12 Registrars	**Model:** ChatGPT-3.5 **Prompt:** Zero-shot **Task:** Rewrite letters for patient understanding while maintaining clinical information	**Comparator:** Human-authored original letters **Design:** Paired before-after study	**Results:** ChatGPT-3.5: FRE 42.0, FKGL 11.3 (Grade 11–12); Control: FRE 57.1, FKGL 9.2 (Grade 9); p < 0.001 ChatGPT-4 produced significantly easier FRE and FKGL than ChatGPT-3.5 (p < 0.001 for both) No significant difference between ChatGPT-4 and human-authored letters for FRE (p = 0.348) or FKGL (p = 0.078)	**Not assessed**	**Information fidelity:** Content remained unchanged; no significant deviations from original; all necessary GP-relevant details retained **Hallucinations:** Emotional embellishments added that were absent from the original letter **Qualitative clinical risk:** Tactless descriptions of patients’ body weight
Roberts et al. (2024) UK	**n = 42** hypothetical letters **Setting:** Aesthetic Surgery (7 procedures: Breast Augmentation/Reduction, Blepharoplasty, Abdominoplasty, Liposuction, Facelift, Rhinoplasty, etc.)	**Models:** ChatGPT-4, ChatGPT-3.5, and Google Bard **Prompt:** Zero-shot **Task:** Generate informed consent letters with complication compliance	**Comparator:** Simulated clinician-authored letters **Design:** Comparative simulation study	**Results:** ChatGPT-4 & Bard: FKGL Grade 9 (Reading age 14–15 years) ChatGPT-3.5: FKGL Grade 8 (Reading age 13–14 years) **Target:** Grade 6 (Not met)	**Not assessed**	**Information fidelity:** Compliance Scoring (% Complication Coverage). ChatGPT-4: BAAPS Score: 0.92; ASPS Website: 0.99; ASPS Consent Bundle; 0.52. ChatGPT-3.5: BAAPS Score: 0.56; ASPS Website: 0.52; ASPS Consent Bundle; 0.39. Google Bard: BAAPS Score: 0.53; ASPS Website: 0.52; ASPS Consent Bundle; 0.30. **Hallucinations:** ChatGPT labelled all complications, including common ones, as “rare” **Qualitative clinical risk:** Not reported
Farooq et al. (2025) UK	**n = 4** scripted consultations **Setting:** Dermatology (Contact Dermatitis, Psoriasis, Leg Ulcer, Rash/Nail) **Note:** Enacted by 2 doctor–patient pairs; audio recorded	**Models:** Heidi AI and ChatGPT-4o **Prompt:** Zero-shot (direct audio input) **Task:** Audio-to-letter (speech-to-text + letter generation)	**Comparator:** Traditional human-authored letters **Design:** Comparative simulation study	**Results:** Heidi: Reading age 1/8 15–18 years, 7/8 18 + years; ChatGPT-4o: Reading age 3/8 15–18 years, 5/8 18 + years; Human: 2/8 12–13 years, 4/8 13–15 years, 2/8 18 + years **Finding:** AI consistently generated more complex text than human controls	**Clinician-Reported Satisfaction** (Two consultant dermatologists): Heidi (4.34/5), ChatGPT-4o (4.59/5), Human (3.52/5)	**Information fidelity:** ChatGPT-4o: 92.6% (Range 69–100%); Heidi: 85% (Range 69–100%); Human: 58.8% (Range 35–85%) **Hallucinations:** Not reported **Qualitative clinical risk:** ChatGPT-4o struggled with spelling drug brand names and generated ‘wordy’ outputs that could benefit from improved paragraphing

AI, Artificial Intelligence; ASPS, American Society of Plastic Surgeons; BAAPS, British Association of Aesthetic Plastic Surgeons; BCC, Basal Cell Carcinoma; CI, Confidence Interval; ENT, Ear, Nose, and Throat; FKGL, Flesch–Kincaid Grade Level; FRE, Flesch Reading Ease; GP, General Practitioner; GU, Genitourinary; H&N, Head and Neck; LLM, Large Language Model; MM, Malignant Melanoma; NHS, National Health Service; PEMAT-P, Patient Education Materials Assessment Tool–Printables; SCC, Squamous Cell Carcinoma; SMOG, Simple Measure of Gobbledygook; UK, United Kingdom.

### Study characteristics

Of the seven included studies, six were conducted in the United Kingdom (UK) [18,19,23–26] and one in Australia [27]. Clinical specialties evaluated included dermatology [18,24], medical oncology [27], orthopaedics [23], hand surgery [26], aesthetic surgery [25], and a multispecialty study of paediatrics, gastroenterology, neurology, psychiatry, renal medicine, gynaecology, respiratory medicine, and ear, nose, and throat surgery [19].

Regarding confidentiality and data governance, two used anonymised real-world clinic data [19,26], whilst the remaining five studies used hypothetical scenarios [18,23–25,27]. All studies used publicly available LLMs. Farooq et al. [24] uniquely assessed a commercial clinical documentation tool (Heidi Health) alongside ChatGPT-4o.

### Risk of bias assessment

Risk of bias was assessed using the Mixed Methods Appraisal Tool (MMAT) 2018 for quantitative non-randomised studies for studies with a comparator group [19,23–26] and quantitative descriptive studies for studies that lacked a comparator group [18,27]. MMAT guidance discourages the calculation of an overall score; therefore, detailed explanations for each domain of included studies are presented in Tables 2 and 3.

**Table 2:** Risk of bias of quantitative non-randomised studies (mixed methods appraisal tool category 3).

Study ID	3.1: Are participants representative of target population?	3.2: Are measurements appropriate regarding both the outcome and intervention (or exposure)?	3.3: Are there complete outcome data?	3.4: Are the confounders accounted for in the design and analysis?	3.5: During the study period, is the intervention administered (or exposure occurred) as intended?
Caterson et al. 2024	No Simulated scenarios, single specialty	No Readability: validated tools Accuracy: clinician Likert scales	Yes	No No mention of the control of any traditional confounding variables in the study’s design or analysis	Yes
Cork & Hopcroft 2025	Yes Real letters (n = 23) across a range of specialties	No Readability: validated tools Patient-reported outcomes: Likert scales	Yes	No No mention of the control of any traditional confounding variables in the study’s design or analysis	Yes
Farooq et al. 2025	No Simulated cases, single specialty	No Readability: validated tools Clinician satisfaction: Likert scale Accuracy: standardised scoring system (unvalidated)	Yes	No No mention of the control of any traditional confounding variables in the study’s design or analysis	Yes
Roberts et al. 2024	No Simulated cases, single specialty	Yes Readability: validated tools Complication compliance: % score of complications included in clinic letters	Yes	No No mention of the control of any traditional confounding variables in the study’s design or analysis	Yes
Stoneham et al. 2024	Yes Real letters (n = 70), new patient consultations in 4-week period, single specialty	No Readability: validated tools Accuracy: assessed by two independent reviewers without a structured framework	Yes	No No mention of the control of any traditional confounding variables in the study’s design or analysis	Yes

**Table 3:** Risk of bias of quantitative descriptive studies (mixed methods appraisal tool category 4).

Study ID	4.1: Is the sampling strategy relevant to address the research question?	4.2: Is the sample representative of the target population?	4.3: Are the measurements appropriate?	4.4: Is the risk of nonresponse bias low?	4.5: Is the statistical analysis appropriate to answer the research question?
Ali et al. 2023	Yes 38 hypothetical scenarios targeted the most common presentations seen by skin cancer specialists	No Hypothetical cases used	Mixed Objective readability indices were used, but other outcomes relied on two clinicians using 10-point Likert scales. No patient-reported outcomes	Yes There is complete reporting of outcome data for all hypothetical letters	Yes Linear regression for error analysis; generalised linear models to evaluate the effect of clinical predictors on median humanness and correctness; ANOVA and Tukey HSD tests to identify statistically significant differences between the cancer groups
Shahnam et al. 2025	Yes 25 hypothetical scenarios covered multiple malignancies, clinical settings, and documentation styles	No Hypothetical cases used	Mixed Objective readability indices and the validated PEMAT-P tool were used to assess readability/understandability, but other outcomes relied on unvalidated clinician/consumer Likert scales	Yes There is complete reporting of outcome data for all hypothetical letters	Yes Descriptive methods and pairwise agreement percentages were appropriate due to skewed data distribution

PEMAT-P, PEMAT-P, Patient Education Materials Assessment Tool–Printables.

All studies reported complete outcome data and administered the intervention as intended. Two studies were judged favourably regarding their representativeness, due to the use of real outpatient clinic letters [19,26]. In contrast, the remaining studies relied on hypothetical cases, which limits external validity [18,23–25,27]. Only one study explicitly reported strategies to control of confounding variables in their design or analysis [18]. Key potential confounders include case complexity, original letter length and structure, patient and reviewer demographics, prompting strategy (e.g., “zero-shot” versus “few-shot”), and presence of medical jargon and abbreviations in original letters or prompts. Only Cork & Hopcroft [19] sampled letters across multiple specialties, whilst most studies were restricted to single specialties and were limited by small sample sizes of letters and/or clinician or patient reviewers. Six of seven studies relied upon Likert scales responses, often determined by a small number of clinicians or patient representatives, instead of employing standardised validated assessment tools.

Taken together, these issues limit the strength of conclusions that can be drawn from our findings.

### Post-hoc simplification for patients

Two studies used LLMs to simplify real existing letters to improve patient understanding, reporting generally null findings.

Cork & Hopcroft [19] inputted existing clinician-authored letters across eight specialties into ChatGPT-4 Classic and asked the tool to convert these letters into language more easily understood by someone with the average UK reading ability. This resulted in a significant reduction in Gunning Fog readability index compared to the original letters (11.35 vs 12.44, p = 0.02), equivalent to a reduction in reading age from 17 to 16 years. However, this remains above the US target reading age of 11–12 years, and no significant differences were observed in other readability indices (FKGL, SMOG, Coleman-Liau, Automated Readability Index). Despite this, 5-point Likert scale responses from 15 patient representatives reported significantly greater understanding of the diagnosis (4.41 ± 0.26 [AI] vs 2.61 ± 0.74 [Control]), understanding of the management plan (4.30 ± 0.41 [AI] vs 2.78 ± 0.62 [Control]), and satisfaction (4.46±0.20 [AI] vs 3.17 ± 0.62 [Control]) compared to the original human-authored letter (all P < 0.0001). All 15 patient representatives preferred the AI-simplified version of the letter, suggesting improvements in readability not detected by most readability indices.

Stoneham et al. [26] employed a similar study design using ChatGPT-3.5 to simplify 70 human-authored hand clinic letters. They found AI made letters significantly more difficult to read, with the FKGL score rising from 9.2 (14–15 years) to 11.3 (16–17 years) (p < 0.001). A significant increase in complexity was also found in FRE scores (reduced from 57.1 to 42.0, p < 0.001), where greater scores indicate greater reading ease. Upgrading the model to ChatGPT-4 produced significantly simpler FRE and FKGL scores relative to ChatGPT-3.5 (p < 0.001 for both), and there was no significant differences between ChatGPT-4 and the original human-authored letters for FRE (p = 0.348) or FKGL (p = 0.078).

These findings regarding formal readability indices suggest LLMs may be unable to adequately simplify clinic letters for the general public. However, these indices do not account for key patient-centred outcomes such as understandability, actionability, and comprehension – and may appear inflated due to the necessary use of medical terms.

Clinical accuracy findings were generally favourable for AI tools. Cork & Hopcroft [19] reported 100% information fidelity with zero loss of clinical information or hallucinations when reviewed by clinicians. Similarly, Stoneham et al. [26] reported all information from original letters was retained; however, authors flagged the hallucination of unprompted emotional embellishments as a potential risk. Regarding qualitative clinical risk, Stoneham et al. [26] reported potential emotional risk due to tactless descriptions of patients’ physical characteristics such as body weight, whilst in Cork and Hopcroft [19] patients significantly preferred AI-generated letters’ language compared to original human-authored letters.

### Generation of clinic letters

Four studies used LLMs to generate new more patient-friendly clinic letters based on hypothetical cases, reporting generally null findings.

One study compared AI-generated letters to traditional dictation methods. Farooq et al. [24] scripted and recorded four dermatological consultations, the recordings of which were inputted into Heidi (https://www.heidihealth.com/) and ChatGPT-4o separately to generate clinic letters. These AI tools generated letters generally at reading ages of 18 + years, with only 3/8 ChatGPT letters rated 15–18 years and 1/8 Heidi letters rated 15–18 (using FRE). This is more complex than the traditional human-authored letters transcribed manually: 2/8 18 + years, 4/8 13–15 years, 2/8 12–13 years.

Among studies without human-authored comparisons, all letters failed to meet the US recommendations of Grade 6 (11–12 years). One study used GPT-3, reporting an FKGL of Grade 8.47 (14 years) [23]. One study used ChatGPT-3.5, reporting FKGL of Grade 8 (13–14 years) [18]. Two studies used ChatGPT-4, with a FKGL of 8.77 (13–15 years) [23] and a SMOG of 8.1 (13–14 years) [27].

However, the limited evidence regarding patient-reported outcomes suggests patients may report high satisfaction with letters despite formal readability indices reporting high complexity.

Shahnam et al. [27] reported Patient Education Materials Assessment Tool for printed materials (PEMAT-P) scores >80% for all AI-generated oncology letters; PEMAT-P is a validated tool to measure the understandability of patient education information, with 80% considered the threshold for high understandability [28]. Furthermore, 72% (90/125) of consumers were willing to receive AI-generated letters based on these examples. However, it was noted that some sentences didn’t flow and sounded robotic, likely attributed to ChatGPT-4o reducing sentence length to meet target readability scores.

Improvements in clinician satisfaction in AI-generated clinic letters have also been documented. Farooq et al. [24] reported better clinician satisfaction on a 5-point Likert scale for both Heidi (4.34/5, range 3.75-5) and ChatGPT-4o (4.59/5, range 3.75-5) compared to traditional human-authored letters (3.52/5, range 2–5). However, this relied on two consultant dermatologist reviewers and patients themselves were not surveyed. Notably, two studies [18,23] did not assess patient-centred outcomes at all, instead relying on clinician judgement of readability, accuracy, and humanness. This represents a critical evidence gap and limits the certainty of our findings.

Regarding information fidelity, Shahnam et al. [27] found 80% (100/125) reviewers agreed that AI-generated patient-directed letters contained all relevant information, rising to 92–98% for physician-directed letters, with only occasional omissions requiring iterative refinement. Farooq et al. [24] reported ChatGPT-4o and Heidi achieved 92.6% and 85% information fidelity respectively, both substantially exceeding traditional human-authored letters (58.8%) in this small scripted scenario study.

Caterson et al. [23] asked three orthopaedic consultants to independently rate information fidelity on a 10-point Likert scale. ChatGPT-4 achieved significantly higher accuracy scores than GPT-3 for both generated clinic letters (8.7/10 vs 7.3/10, p = 0.024) and predicted management plans (7.9/10 vs 6.8/10, p < 0.001). However, the same study found LLMs sometimes hallucinated incorrect or inappropriate information about the management plan (e.g., when casts will be removed), but the potential impact on patient safety was not evaluated [23].

Ali et al. [18] reported the greatest variation in accuracy. Despite a median fidelity rate of 70%, this ranged from 10-90%, with fidelity and hallucination rates varying significantly between case types.

Regarding qualitative clinical risk, three of four studies presented difficulties including tactless descriptions of patients’ body weight [23], misspelling of drug brand names [24], and patients’ perceptions of AI-generated letters to be robotic or less empathetic [27]. Ali et al. [18] was the only study to quantify the humanness of letters; this was assessed via 10-point Likert scales completed by two independent clinicians with a median of 70% (range 50%-90%) – although patient views were not assessed by this study.

Across studies, there was a high degree of variability in accuracy outcomes; however, the severity of inaccuracies, and therefore implications on patient care, are poorly reported.

A key proposed benefit of AI-generated clinic letters is time-saving and improved efficiency. Farooq et al. [24] uniquely assessed this outcome, reporting that AI-generated clinic letters were generated significantly faster (Heidi 27 seconds, ChatGPT-4o 54 seconds) compared to traditional dictation methods (10 minutes 54 seconds). This represents a > 10-fold time saving, with potential implications for clinician workload and operational efficiency.

### Enhancement of existing letters

Only one study, Roberts et al. [25], used LLMs to enhance existing letters by incorporating complication and consent profiles. The study evaluated three AI models, all of which produced letters exceeding the recommended limit when evaluated using the FKGL: Grade 9 for ChatGPT-4 and Bard (age 14–15), and Grade 8 for ChatGPT-3.5 (age 13–14). The study did not assess patient-centred outcomes.

Regarding information fidelity, Roberts et al. [25] reported variable compliance in incorporating complication profiles recommended by surgical guidelines: ChatGPT-4 compliance ranged from 0.52-0.92; ChatGPT-3.5 compliance ranged from 0.39-0.56; Google Bard compliance ranged from 0.30-0.53. Furthermore, Roberts et al. [25] identified a critical clinical risk: ChatGPT misclassified all complications (including wound infection, occurring in 16% of breast reductions) as ‘rare’, whereas European guidelines classify this frequency as ‘very common’.

These findings regarding enhancement of clinic letters are based on one study so should be treated with caution; more studies are required to sufficiently inform conclusions.

## Discussion

### Key findings

This systematic review identified seven studies evaluating the use of AI and LLMs to generate, simplify, and enhance outpatient clinic letters. Overall, the evidence base remains small, heterogeneous, and subject to substantial bias. Nevertheless, several preliminary findings emerge.

First, AI-assisted clinic letters demonstrated generally high average information fidelity, often comparable to or exceeding that of human-authored letters. However, this ranged from 10% to 90% in Ali et al. [18], and 30% to 52% for the consent bundle in Roberts et al. [25]. There was also a recurring risk of hallucinated embellishments or incorrect or tactless additions, particularly where AI introduced guidance absent from the source consultation or prompt. The potential for patient harm from these areas was not evaluated in any study, thus necessitating clinician oversight and precluding autonomous deployment of AI-generated letters in current practice.

Second, patient-reported comprehension and satisfaction were high in the two studies that assessed patient-centred outcomes, although this should be treated with caution due to the limitations discussed below. Notably, these improvements occurred despite AI-generated letters failing to meet recommended readability thresholds using standard indices. This suggests that conventional readability metrics may not fully capture how patients experience and understand clinic correspondence, and that AI may improve clarity through mechanisms not adequately reflected by automated formulae alone.

Third, AI tools may offer a substantial efficiency advantage, with one study demonstrating more than a ten-fold reduction in time required to generate clinic letters. While evidence for this outcome is currently sparse, the potential implications for clinician workload and service efficiency are significant and warrant further investigation.

### Implications for practice and policy

These findings have several implications for healthcare systems seeking to improve patient-centred communication. First, whilst the current evidence may be interpreted as generally positive, the quantity and level of evidence is insufficient to support AI and LLM use for clinic letters presently. Importantly, no evidence supports unsupervised AI-generated letters being sent directly to patients, given ongoing risks of hallucination, inappropriate advice, and deviation from clinical intent.

Second, careful selection of LLM model to generate letters is important. Within study comparisons are limited but displayed significant variability between models for readability and accuracy outcomes. As new models are developed and updated, their performance may change unpredictably in different contexts, and further research is required to evaluate these changes. Additionally, key limitations in generic AI models without healthcare-specific training or access to paywalled research articles have been noted [25,26]. Various ambient AI scribes have been developed for use specifically in healthcare settings. Whilst these tools promise benefits for reduced clinician workload, concerns about privacy, hallucinations, and bias in training data persist [29] These are being evaluated in an ongoing systematic review [30]. Beyond one study using Heidi Health [24], these models were not evaluated in this current review due to the lack of studies investigating their use in outpatient clinic letters. We recommend this area as an avenue for future research.

Third, AI-assisted letter simplification may help address longstanding barriers to implementing the AoMRC (2018) and NHS England (2023) guidance recommending that outpatient clinic letters should be written directly to patients [3,4]. Despite evidence that simplified, patient-directed letters improve both patient comprehension [16] and satisfaction [31], uptake has been limited by time constraints, limited training, and a lack of awareness of AoMRC guidance [17]. Backed by greater future evidence, AI-supported workflows could enable scalable implementation of these policies, provided that robust governance, monitoring, and safety frameworks are in place.

Fourth, AI offers a potential solution to clinicians’ concerns that simplifying letters for patients may lose critical details for the patient’s GP [16]. Indeed, studies in this review noted occasional omissions in AI-simplified letters [23,24,27]. A dual-letter approach, whereby the secondary care physician writes a clinically detailed letter addressed to the GP, whilst AI generates a simplified patient letter alongside this, could address this tension. While clinician-written dual letters have shown benefit [16], no study has yet evaluated AI-generated dual correspondence, representing an important opportunity for future research.

### Relationship to existing evidence on clinic letter readability

Previous studies have shown clinic letters are consistently written at a reading level above that of the general population [5]. Additionally, clinician-led interventions have failed to significantly improve readability indices to meet US guidelines of Grade 6 (11–12 years) [14,15], despite improvements in patient-reported metrics [15]. These findings mimic those in AI-generated clinic letters, thus raising questions about the validity of using readability indices as proxies for patient understanding in the context of clinic letters.

Readability formulae heavily rely on sentence length and word complexity [32], yet medical language frequently includes unavoidable multisyllabic terms [33]. Attempts by AI models to optimise these metrics may lead to shorter, less natural sentences, which patients may perceive as robotic or less empathetic letters [27]. These limitations suggest that future evaluations should prioritise patient-centred outcomes alongside, rather than subordinate to, automated readability measures.

### Quality of evidence and certainty assessment

Using the GRADE criteria, the overall certainty of evidence across all five outcomes domains was rated as ‘Very Low’. All included studies were observational/ cross-sectional, so certainty for each outcome initially rated as ‘Low’. Publication bias could not be assessed for any outcomes due to the low study volume (<10). No criteria were fulfilled for upgrading the certainty of findings for each outcome. Assessments across each domain are detailed below and in Table 4.

- Formal readability metrics (7 studies) were downgraded to ‘Very Low’ due to serious inconsistency (wide variability in reading levels across studies), indirectness (5/7 studies used hypothetical cases), and imprecision (small sample sizes). Risk of bias was rated as ‘not serious’ due to objective algorithmic scoring.
- Patient-reported outcomes (2 studies) were downgraded to ‘Very Low’ due to a serious risk of bias (due to selection bias and subjective Likert scores), indirectness (use of hypothetical cases in Shahnam et al. [27]), and imprecision (only two studies). Results were consistent between studies, but these limitations reduce the certainty of conclusions.
- Information fidelity (6 studies) was downgraded to ‘Very Low’ due to a serious risk of bias (uncontrolled confounders such as case complexity), inconsistency (fidelity ranging from 10% to 100%), indirectness (4/6 studies used hypothetical cases), and imprecision (small sample sizes).
- AI hallucination rate (5 studies) was downgraded to ‘Very Low’ due to serious risk of bias (unstandardised observer reporting), inconsistency in results, indirectness (3/5 studies used hypothetical cases), and imprecision (small sample sizes).
- Qualitative clinical risk (6 studies) was downgraded to ‘Very Low’ due to serious risk of bias (unstandardised observer reporting or unvalidated rating scales), inconsistency in results, indirectness (4/5 studies used hypothetical cases), and imprecision (small sample sizes).

**Table 4:** GRADE evaluation of certainty for each primary outcome.

No. of studies	Study Designs	Risk of Bias	Inconsistency	Indirectness	Imprecision	Publication Bias	Certainty of Evidence
**Formal readability indices (e.g., Flesch Reading Ease, Flesch-Kincaid Grade Level)**							
**7**	Observational/ Cross-Sectional	**Not Serious** Standardised algorithmic readability indices.	**Serious** Substantial variability in reading level of AI outputs.	**Serious** Five of seven studies used hypothetical cases.	**Serious** Few studies with small sample sizes.	Unable to calculate*	**Very Low**
**Patient-reported outcomes**							
**2**	Observational/ Cross-Sectional	**Serious** Potential selection bias within the homogenous target consumer feedback panels.	**Not Serious** Patients reported high readability of the AI-simplified letters.	**Serious** One of two studies used hypothetical cases.	**Serious** Few studies with small sample sizes.	Unable to calculate*	**Very Low**
**Information fidelity**							
**6**	Observational/ Cross-Sectional	**Serious** Confounders (e.g., case complexity) were not controlled	**Serious** Fidelity ranged from 10-100%, with some studies noting dangerous omissions.	**Serious** Four of six studies used hypothetical cases.	**Serious** Few studies with small sample sizes.	Unable to calculate*	**Very Low**
**AI hallucinations**							
**5**	Observational/ Cross-Sectional	**Serious** Unstandardised observer reporting	**Serious** One study reported zero hallucinations, with others noting inaccurate additions to letters	**Serious** Three of five studies used hypothetical cases.	**Serious** Few studies with small sample sizes.	Unable to calculate*	**Very Low**
**Qualitative clinical risk**							
**6**	Observational/ Cross-Sectional	**Serious** Unstandardised observer reporting	**Serious** Variable reporting including misspellings, tactless descriptions of patients, robotic tone	**Serious** Four of six studies used hypothetical cases.	**Serious** Few studies with small sample sizes.	Unable to calculate*	**Very Low**

AI, Artificial intelligence; No., Number. *The small number of included studies (seven) prevented the formal assessment of publication bias.

### Limitations and recommendations for future research

Several limitations reduce the generalisability of findings. Most notably, five of the seven included studies used purely hypothetical or scripted scenarios, rather than real clinical encounters or letters. Whilst this protects against any confidentiality breaches, it limits external validity of findings; hypothetical cases may lack the nuance and complexity of real cases, thus potentially overestimating AI performance. Future studies should include a range of real cases across a variety of specialties and complexities that represent real clinical practice, with the utmost attention to anonymising any patient-identifying details. Additionally, greater reporting and analysis of confounding factors is required, as this was a limitation of all included studies.

Patient-centred outcomes remain under-represented in clinic letter research. Patient comprehension was assessed in only two studies which carry individual limitations. Cork and Hopcroft [19] compared AI-generated to human-authored letters, but relied on the subjective responses of only 15 patient representatives. Whilst Shahnam et al. [27] used the validated PEMAT-P scores to assess understandability, this study failed to include clinician-authored comparators. Studies reporting clinician-centred outcomes had similar limitations. Future evaluations should embed patient and public involvement, incorporate validated comprehension tools, and use mixed methods approaches to better capture patient experience.

Implementation outcomes also require further investigation. Beyond time savings, future work should examine costs, workflow integration, training requirements, and long-term sustainability, as well as impacts on GP satisfaction, healthcare utilisation, and clinical outcomes. The potential impact of information omissions, hallucinations, and inappropriate advice on patient safety is also an important consideration for implementation. Long-term follow up at sites piloting such AI tools will support this, and the effects of dual-letter approaches on GP satisfaction may also be explored to address concerns about loss of clinical information.

Further research should also be conducted into the development and validation of healthcare-specific LLMs. Current evidence suggests general-purpose LLMs, despite being explored in all included studies, have limited appreciation of the nuance of medical language and guidelines. Additionally, confidentiality safeguards must be integrated into LLMs to prevent data breaches and ensure patient confidence in such tools. However, even with these safeguards, it is important from an ethical perspective to ensure patients are adequately informed about the processing of their data and appropriate consent processes are in place subject to local/national recommendations (e.g., an opt-in or opt-out system).

Furthermore, whilst referred to as “LLMs”, the commercial tools used in included studies should be more correctly described as LLM-driven products with largely unreported pre- and post-processing which may influence their outputs. The continual evolution of such systems limits the reproducibility of study findings and the quality of conclusions. Repeating studies using an open-weight LLM (where parameters learned during training are publicly reported) may help to address such limitations, and this transparency should be encouraged in the development of healthcare-specific LLMs.

Finally, research should explore AI’s potential to reduce health inequalities. AI-simplified clinic letters perhaps have the greatest potential for benefit in patients with limited health literacy, which itself is associated with poorer clinical outcomes [34]. Only two existing studies involved patient representatives, and the only study that reported their demographics lacked representation of the general population [19]. Of 15 participants, 13 were White/Caucasian, 12 had qualifications beyond secondary education, nine were ≥65, and all spoke English as their first language [19]. Patients who do not speak English as a first language are documented to have poorer engagement with clinic letters [35], so may also be key beneficiaries of AI-simplified or translated letters. Future research must prioritise patient and public involvement and the views of patient representatives from diverse backgrounds to evaluate potential impacts on health equity.

This present systematic review is limited by inconsistent design and outcome reporting of the limited studies that have been conducted in this field, thus preventing meta-analysis and the certainty of conclusions. A formal assessment of publication bias was also not possible; this is important as novel fields are susceptible to over-reporting of positive findings and under-reporting of negative or unsafe findings. Future studies should employ a comprehensive battery of assessed outcomes and account for the limitations previously described. Regularly updated systematic reviews with formal meta-analyses and publication bias assessments are required to continually evaluate the rapid evolution of LLMs and their use in clinic letters.

## Conclusion

In summary, preliminary evidence suggests AI and LLMs used to support the generation and simplification of outpatient clinic letters may lead to potential benefits for clinical accuracy, patient understanding, and efficiency. However, current evidence is insufficient to recommend routine clinical use beyond experimental contexts, and significant gaps remain regarding safety, equity, and implementation. Large, controlled, patient-centred, real-world studies are urgently needed to define the appropriate role of AI in written clinical communication.

## Supporting information

S1 Table PRISMA checklist. (DOCX)

S2 Table Search queries for each database. (DOCX)

S3 Table Completed data extraction spreadsheets. (XLSX)

S4 Table Excluded studies with reasons. (XLSX)
