# Supplementary material for: Large language models and artificial intelligence for generating, simplifying, and enhancing outpatient clinic letters: A systematic review
Source: PLOS Digit Health. 2026 Sep 22;5(9):e0001745. doi: 10.1371/journal.pdig.0001745 (PMC13596789; doi:10.1371/journal.pdig.0001745)
Supplement: Supplementary file 2 [file pdig.0001745.s002.docx]

**S2 Table: Search Query for Embase, PubMed, CENTRAL, CINAHL, and Web of Science.**

| **Database** | **Search Query** |
| --- | --- |
| **Embase** | (("letters" or "doctor-patient communication" or "clinic letter*" or "clinical letter*" or "outpatient letter*" or "consultation letter*" or "medical letter*" or "patient letter*" or "clinic correspondence" or "outpatient communication" or "written communication" or "clinical correspondence" or "patient communication" or "clinic note*") and ("artificial intelligence" or "natural language processing" or "machine learning" or "deep learning" or "large language model*" or ChatGPT or "GPT-3" or "GPT-4" or "Claude" or "Copilot" or "Grok" or "Gemini" or "Deepseek" or "Med-PaLM" or "BioGPT" or "BERT" or "T5" or "generative AI" or "transformer model*" or "text generation" or "text simplification" or "automated documentation" or "language model*" or "automatic report generation") and ("simplif*" or "readability" or "comprehension" or "drafting" or "patient-friendly" or "plain language" or "clarity" or "quality improvement" or "accuracy" or "fidelity" or "patient understanding" or "summaris*" or "satisfaction" or "Flesch-Kincaid" or "SMOG" or "Flesch Reading Ease" or "Fog index" or "Dale-Chall")).ab,ti. |
| **PubMed** | ( (letters[Mesh] OR doctor-patient communication[Mesh] OR "clinic letter*"[tiab] OR "clinical letter*"[tiab] OR "outpatient letter*"[tiab] OR "consultation letter*"[tiab] OR "medical letter*"[tiab] OR "patient letter*"[tiab] OR "clinic correspondence"[tiab] OR "outpatient communication"[tiab] OR "written communication"[tiab] OR "clinical correspondence"[tiab] OR "patient communication"[tiab] OR "clinic note*"[tiab]) ) AND ( "artificial intelligence"[Mesh] OR "natural language processing"[Mesh] OR "machine learning"[Mesh] OR "deep learning"[tiab] OR "large language model*"[tiab] OR ChatGPT[tiab] OR "GPT-3"[tiab] OR "GPT-4"[tiab] OR "Claude"[tiab] OR "Copilot"[tiab] OR "Grok"[tiab] OR "Gemini"[tiab] OR "Deepseek"[tiab] OR Med-PaLM[tiab] OR BioGPT[tiab] OR BERT[tiab] OR T5[tiab] OR "generative AI"[tiab] OR "transformer model*"[tiab] OR "text generation"[tiab] OR "text simplification"[tiab] OR "automated documentation"[tiab] OR "language model*"[tiab] OR "automatic report generation"[tiab] ) AND ("simplif*"[tiab] OR "readability"[tiab] OR "comprehension"[tiab] OR "drafting"[tiab] OR "patient-friendly"[tiab] OR "plain language"[tiab] OR "clarity"[tiab] OR "quality improvement"[tiab] OR "accuracy"[tiab] OR "fidelity"[tiab] OR "patient understanding"[tiab] OR "summaris*"[tiab] OR "satisfaction"[tiab] OR "Flesch-Kincaid"[tiab] OR "SMOG"[tiab] OR "Flesch Reading Ease"[tiab] OR "Fog index"[tiab] OR "Dale-Chall"[tiab]) |
| **CENTRAL** | (("letters" or "doctor-patient communication" or clinic letter* or clinical letter* or outpatient letter* or consultation letter* or medical letter* or patient letter* or "clinic correspondence" or "outpatient communication" or "written communication" or "clinical correspondence" or "patient communication" or clinic note*) and ("artificial intelligence" or "natural language processing" or "machine learning" or "deep learning" or large language model* or ChatGPT or "GPT-3" or "GPT-4" or "Claude" or "Copilot" or "Grok" or "Gemini" or "Deepseek" or "Med-PaLM" or "BioGPT" or "BERT" or "T5" or "generative AI" or transformer model* or "text generation" or "text simplification" or "automated documentation" or language model* or "automatic report generation") and (simplif* or "readability" or "comprehension" or "drafting" or "patient-friendly" or "plain language" or "clarity" or "quality improvement" or "accuracy" or "fidelity" or "patient understanding" or summaris* or "satisfaction" or "Flesch-Kincaid" or "SMOG" or "Flesch Reading Ease" or "Fog index" or "Dale-Chall")):ti,ab,kw |
| **CINAHL** | (("letters" or "doctor-patient communication" or "clinic letter*" or "clinical letter*" or "outpatient letter*" or "consultation letter*" or "medical letter*" or "patient letter*" or "clinic correspondence" or "outpatient communication" or "written communication" or "clinical correspondence" or "patient communication" or "clinic note*") and ("artificial intelligence" or "natural language processing" or "machine learning" or "deep learning" or "large language model*" or ChatGPT or "GPT-3" or "GPT-4" or "Claude" or "Copilot" or "Grok" or "Gemini" or "Deepseek" or "Med-PaLM" or "BioGPT" or "BERT" or "T5" or "generative AI" or "transformer model*" or "text generation" or "text simplification" or "automated documentation" or "language model*" or "automatic report generation") and ("simplif*" or "readability" or "comprehension" or "drafting" or "patient-friendly" or "plain language" or "clarity" or "quality improvement" or "accuracy" or "fidelity" or "patient understanding" or "summaris*" or "satisfaction" or "Flesch-Kincaid" or "SMOG" or "Flesch Reading Ease" or "Fog index" or "Dale-Chall")) |
| **Web of Science** | (("letters" or "doctor-patient communication" or clinic letter* or clinical letter* or outpatient letter* or consultation letter* or medical letter* or patient letter* or "clinic correspondence" or "outpatient communication" or "written communication" or "clinical correspondence" or "patient communication" or clinic note*) and ("artificial intelligence" or "natural language processing" or "machine learning" or "deep learning" or large language model* or ChatGPT or "GPT-3" or "GPT-4" or "Claude" or "Copilot" or "Grok" or "Gemini" or "Deepseek" or "Med-PaLM" or "BioGPT" or "BERT" or "T5" or "generative AI" or transformer model* or "text generation" or "text simplification" or "automated documentation" or language model* or "automatic report generation") and (simplif* or "readability" or "comprehension" or "drafting" or "patient-friendly" or "plain language" or "clarity" or "quality improvement" or "accuracy" or "fidelity" or "patient understanding" or summaris* or "satisfaction" or "Flesch-Kincaid" or "SMOG" or "Flesch Reading Ease" or "Fog index" or "Dale-Chall")) |
